# Design of Scalable Optical Decoder based on Hexagonal Plasmonic Modes induced on Topological Insulator Surface States

**DOI:** 10.1038/s41598-019-45607-z

**Published:** 2019-06-24

**Authors:** Siddharth Srivastava, Priyanshu Jain, Tanmoy Maiti

**Affiliations:** Plasmonics and Perovskites Laboratory, Department of Materials Science and Engineering, IIT Kanpur, U.P. 208016 India

**Keywords:** Optical data storage, Nanophotonics and plasmonics, Nanophotonics and plasmonics, Nanophotonics and plasmonics

## Abstract

In the present work, optical decoder based on hexagonal plasmonic lens encrypted on topological insulator is designed. Using Finite Difference Time Domain (FDTD) simulation we have shown 2D optical lattice of scalar vortices in hexagonal plasmonic lens using surface states of topological insulator (Bi_1.5_Sb_0.5_Te_1.8_Se_1.2_). To ensure feasible and flexible physical dimensions, scaling of the optical device is proposed via increasing area density of vortices. This is numerically obtained by changing radius of hexagonal lens or decreasing incident wavelength. Using these scalable optical vortex lattices, a device scheme is proposed for storing or decoding information. Advantage of scaling in optical devices without any additional processing step shows the promise of this technology for future devices. Simulation results are further validated by detailed theoretical calculation of electric field intensity and phase distribution.

## Introduction

The field of plasmonics and metamaterials has grown immensely in the recent past. The wide range of applications of plasmonics include light harvesting^1^, biological sensors for pregnancy tests^2^, data carriers for optical data processing chips^3^ and ultra-compact interconnects^4,5^. However, the choice of materials poses a limitation. Noble metals such as Au and Ag have been utilized, but they suffer from the problems of inter-band electronic transitions and Drude losses^6^, which hampers device practicality. Therefore, energy dissipation in plasmonic media has hindered commercialization of plasmonic devices, which are based on surface plasmon polariton (SPP) propagation. The losses are magnified in the high frequency ranges, such as in ultraviolet (UV) range. This has motivated a search for low-loss media for such applications. In that line of effort, advancement has been made in infrared (IR) based plasmonic devices, such as graphene, conductive oxides, and nitride-based devices^7,8^. However, the search is still on for UV-range applications^7^ where the loss in existing materials is very large for device application. Recently it has been reported that optical access is possible in the surface states of ‘topological insulators’ (TI)^9^ which show localized surface plasmons in visible-UV spectral range. TI, such as Bi_2_Se_3_^10^ and Bi_1.5_Sb_0.5_Te_1.8_Se_1.2_ (BSTS)^11^, are materials that exhibit conductive behavior at the surface and insulating behavior in the bulk interior, such that electronic motion is confined to the surface. Recently it has been reported based on DFT calculation^11,12^ that figure of merit (FOM) of SPP propagation is better for TI than that of noble metals like Au, Ag in UV-vis range, due to much lower loss at high frequencies for TI. Hence, the following work will focus solely on TI for plasmonic applications in UV-vis range, where the noble metals do not perform well.

In the present work, first we have numerically calculated the E-field intensity and phase distribution of the emission from a hexagonal plasmonic lens (HPL) encrypted on TI in visible-UV range using FDTD simulation. 2-D optical lattices of scalar vortices are obtained in HPL. Moreover, we have validated the FDTD results by theoretical calculations. Scaling has been the driving force in electronic industries over the past several decades for downsizing devices. Inspired by the scaling of devices in electronics industry over the decades^13^, in the present work we have proposed the scaling of optical information decoding devices by changing the size of the hexagonal lens, as well as incident wavelength. Further, we have proposed a novel design of device using HPL. To the best of our knowledge, this is the first report of utilizing scaling behavior of lattice of scalar vortices. Furthermore, we have shown the feasibility of the hexagonal plasmonic device structure by calculating the E-field in the far field.

## Design Principle

**Figure 1:**
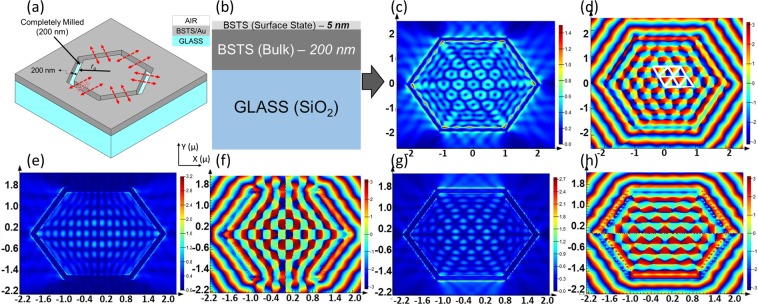
(**a**) Isometric view - Hexagonal plasmonic lens: red arrows indicate direction of SPP propagation; (**b**) Device configuration; FDTD results for BSTS/Glass $({{\rm{r}}}_{{\rm{o}}}=5{{\rm{\lambda }}}_{{\rm{spp}}})$– (**c**,**d**) Intensity and Phase for RCP illumination, (**e**,**f**) Intensity and Phase for X-polarized illumination, (**g**,**h**) Intensity and Phase for Y-polarized illumination.

Here, we have designed a bilayer plasmonic metasurface to operate at visible-UV frequencies. The focusing properties and surface plasmon confinement have been evaluated for TI/substrate plasmonic lenses in the near field as well as in the far field. Silica glass has been used as substrate. The TI layers have been milled completely through their thickness of 205 nm, in the shape of a hexagon as shown schematically in Fig. 1(a). The difference between inner and outer radii of the hexagon grating has been kept at 20 nm. The radii (perpendicular distance of sides from center) have been taken to be ${r}_{o}=3{\lambda }_{spp},4{\lambda }_{spp},5{\lambda }_{spp,}$where *λ*_*spp*_ is the effective wavelength of SPP. The device is studied in transmission mode.

It is reported^14^ that optical lattices form because of superposition of several surface plasmon beams. Here, the etched hexagonal slit has been utilized to excite multiple coherent plasmonic waves. When a polarized beam is incident on the device in transmission mode, it causes the sharp corners of each slit to produce numerous plasmonic waves. These surface waves propagate away from the edges, as shown by the red arrows in Fig. 1(a). The surface plasmonic waves moving towards the center superpose to form polygonal plasmonic modes, as shown in Fig. 1 (c)–(h).

## Results and Discussion

In the current investigation, BSTS has been chosen as the TI material, in which the contributions of the surface states and bulk states are treated differently, based on previously reported DFT analyses^11,12^ and experimental inferences. This has been manifested via a TI-slab model in our simulations. In the TI-slab model, the BSTS compounds contain topological surface states that exhibit a Drude-like dispersion, while the bulk state exhibits Tauc-Lorentz dispersion^11^. Hence, in our simulation and theoretical analysis, we have assumed the BSTS layer in the device to consist of 5 nm surface state and 200 nm bulk state, as shown in Fig. 1(b). The complex dielectric constants are different for the two layers^11,12^. Further, to generalize our theory we used other TI, like Bi_2_Se_3_ and got similar results.

Using FDTD simulation E-field and phase profiles are calculated for BSTS/Glass devices. We have used illumination of wavelength 415 nm. Correspondingly-

$${{\rm{\lambda }}}_{{\rm{spp}}}(\frac{{\rm{BSTS}}}{{\rm{Air}}})=405.21\,{\rm{nm}}.$$

Figure 1 presents results TI samples for near-field RCP, X-polarized and Y-polarized illumination. The device has radius r_o_ = 5λ_spp_.

Under RCP illumination, in Fig. 1 (c),(d), we obtain a 2-Dimensional optical lattice of scalar vortices. The lattice constant of the optical lattice – defined as distance between singularity points of adjacent vortices – is calculated as ~490 nm for BSTS. Such scalar vortices have numerous applications including optical trapping, optical data storage^14^, faster quantum computing, optical tweezers, and telecommunications^15^. In the present work, we specifically focus on the application of scalar vortices for optical information decoder.

### Scaling behavior

**Figure 2:**
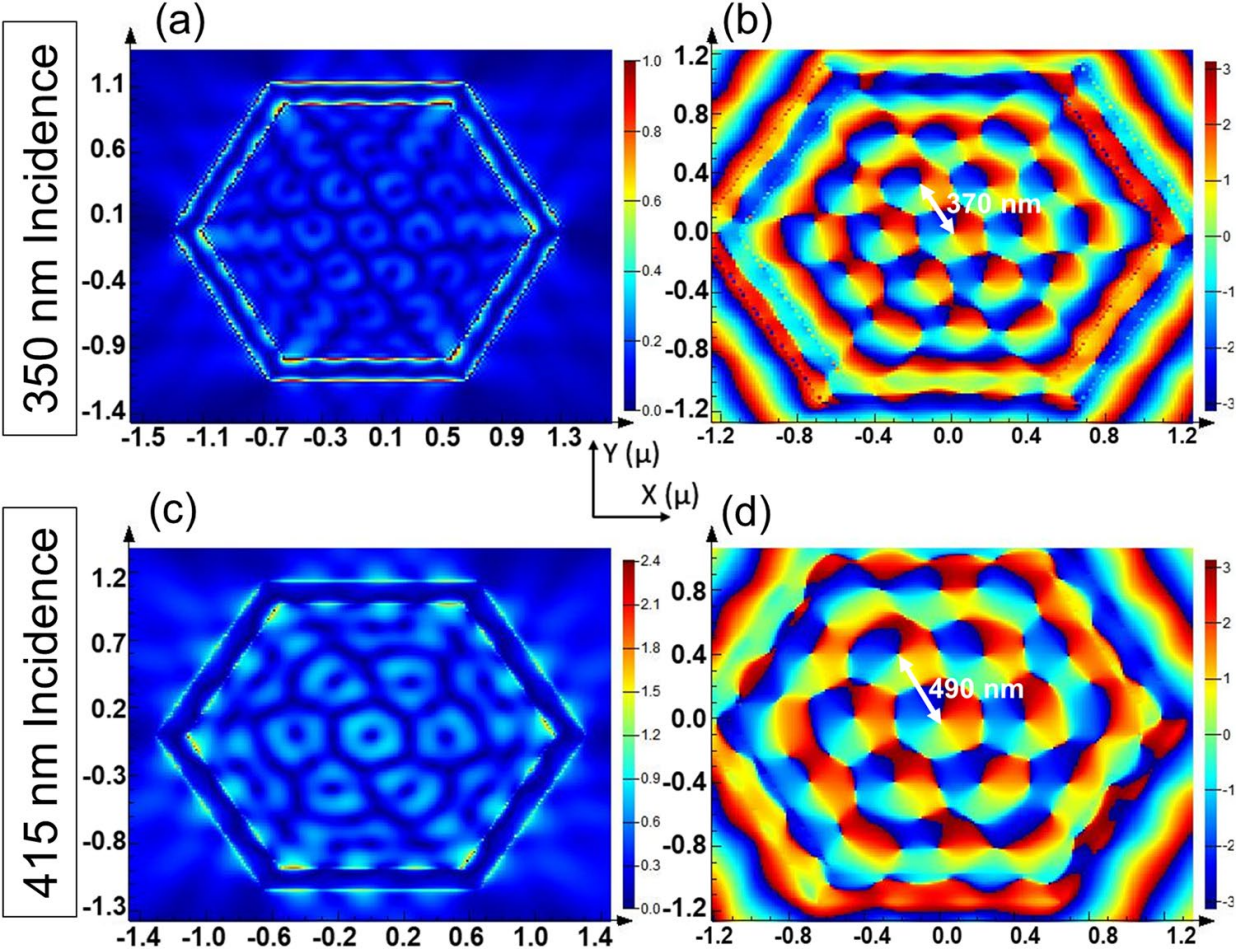
FDTD results for RCP incidence of variable wavelengths for BSTS/Glass.

Scaling of devices has been perceived as a very important parameter for greater performance and portability in the electronics industry. We have used the analogy to explore the feasibility of scaling in proposed optical decoder device. We have shown that device scaling of such TI-based optical devices can be realized by varying incident wavelength. We have observed, in Fig. 2, that by decreasing the wavelength of incident beam, we can decrease the lattice constant of the 2D optical vortex lattice. This results in a higher area-density of optical vortices. For example, altering the wavelength of illumination from 415 nm to 350 nm causes the change in lattice constant from 490 nm to 370 nm. Hence, an extra outer ring of vortices is generated meaning the number of vortices increase from 7 to 19. This observation provides an additional option for scaling a memory cell or nanoscale decoders. Moreover, it opens up possibilities of engineering layered or hidden optical memories.

**Figure 3:**
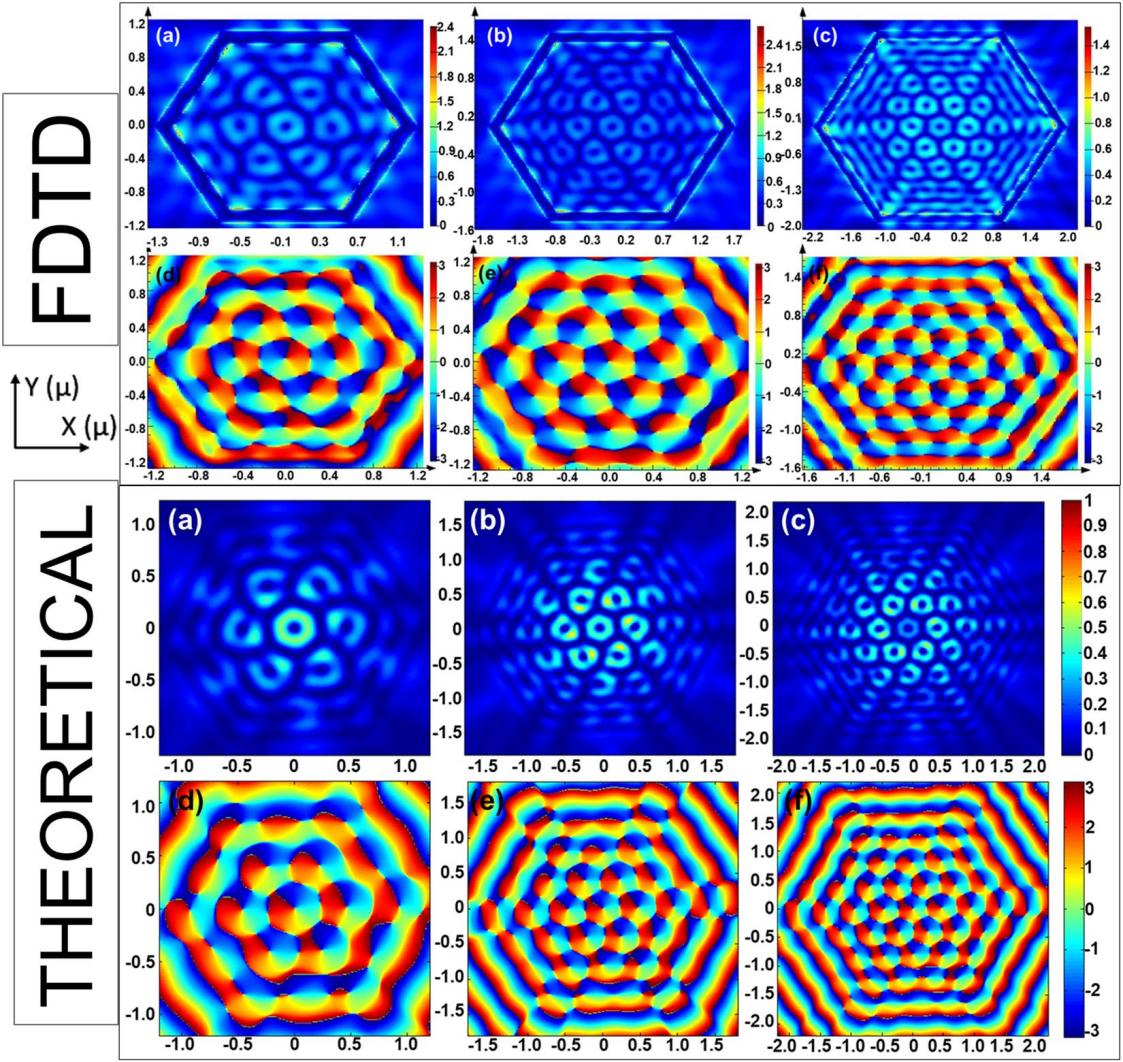
FDTD simulation and Theoretical Calculations of E-field (E_z_) distribution (**a**,**b**,**c**) and phase distribution (**d**,**e**,**f**) for Hexagonal Plasmonic Lens under RCP illumination with varying radii, ${{\rm{r}}}_{{\rm{o}}}=({\rm{a}},{\rm{d}})\,3{{\rm{\lambda }}}_{{\rm{spp}}},({\rm{b}},{\rm{e}})\,4{{\rm{\lambda }}}_{{\rm{spp}}},({\rm{c}},{\rm{f}})\,5{{\rm{\lambda }}}_{{\rm{spp}}}$, for BSTS/Glass.

We also obtained scaling behavior by varying hexagonal grating size of the HPL. FDTD simulation and MATLAB plot of theoretical calculation shown in Fig. 3, respectively present the E-field intensity and phase pattern observed for different radii of hexagonal grating on BSTS sample, ${{\rm{r}}}_{{\rm{o}}}=3{{\rm{\lambda }}}_{{\rm{spp}}},4{{\rm{\lambda }}}_{{\rm{spp}}},5{{\rm{\lambda }}}_{{\rm{spp}}}$, under RCP illumination. We have observed that on incrementing the radius by λ_spp_, the number of vortices formed in the interior region of the hexagonal lens increases parabolically. In fact, if nλ_spp_ is the radius of the hexagonal lens, then the number of vortices follows the relation: [3(n − 1)(n − 2) + 1].

Hence, simply incrementing the lens radius by λ_spp_ can lead to an increase in the area density of 2D optical lattice of vortices. This ‘scaling behavior’ has been further validated using theoretical calculations as discussed below. We propose that such a behavior can be utilized for memory decoding applications, wherein each vortex is utilized for the purpose of reading information. This is particularly exciting because of the simplicity of the device scaling.

**Figure 4:**
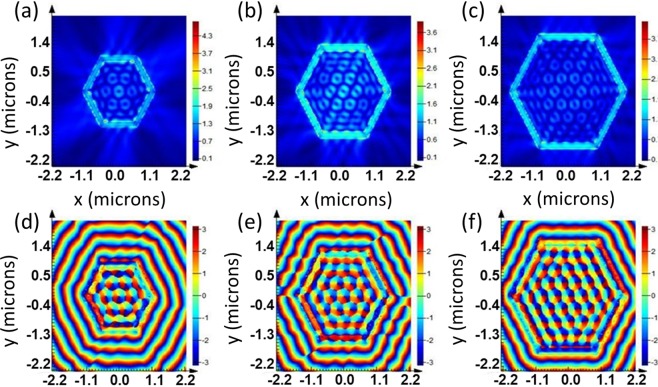
FDTD simulation of Electric field (E_z_) distribution (**a**,**b**,**c**) and phase distribution (**d**,**e**,**f**) for Bi_2_Se_3_/Glass Hexagonal Plasmonic Lens under RCP illumination with varying radii, ${{\rm{r}}}_{{\rm{o}}}=3{{\rm{\lambda }}}_{{\rm{spp}}},4{{\rm{\lambda }}}_{{\rm{spp}}},5{{\rm{\lambda }}}_{{\rm{spp}}}$.

Furthermore, FDTD simulation has been carried out for the E-field intensity and phase profiles for LCP, x and y polarized incident beams presented in Supplementary Figures S1, S2 and S3. A ‘scaling behavior’ can clearly be observed in all the cases. Also, similar scaling was observed in another TI, for example Bi_2_Se_3_, as shown in Fig. 4.

### Analytical study of hexagonal plasmonic lens

**Figure 5:**
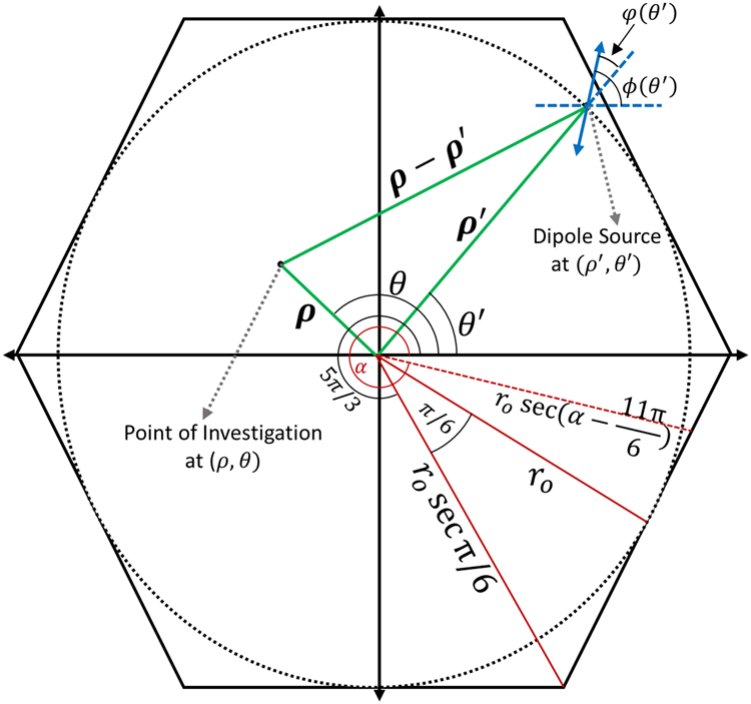
Schematic presenting terms in the equations; In red is the [5π/3,2π] section of hexagon.

To validate our FDTD results, we have theoretically calculated the E-field intensity, as well as the phase of the output beam from HPL. All succeeding theoretical calculations have been made for near field, i.e. on the device surface, for simplicity. For a circular plasmonic lens (CPL), the equation of a plasmonic field at an observation point (ρ, θ) due to the excitation along an incremental length of a slit is given by^16^:

$$d{E}_{z}(\rho ,\theta ,z)=A(\varphi (\theta ^{\prime} )){e}^{-{k}_{a}z}{e}^{j\omega (\varphi (\theta ^{\prime} ),\theta ^{\prime} )}{e}^{j{k}_{spp}|\rho -\rho ^{\prime} |}d\theta ^{\prime} $$

where, *ρ*, *θ*, *z*- radial, azimuthal and z-direction coordinates of the E_z_ at a general point of observation; (*ρ*′, *θ*′,) - coordinates of dipole sources; k_a_ - attenuation coefficient in z-direction; |*ρ* − *ρ*′| - distance between source of SPPs and point of investigation; *ϕ*(*θ*′) - azimuthal distribution function of dipole orientations with respect to x-axis; $\phi (\theta ^{\prime} )$ - azimuthal distribution function of the dipole orientations with respect to radial vector, i.e. $\phi (\theta ^{\prime} )=\varphi (\theta ^{\prime} )-\theta ^{\prime}$. For better visualisation, these terms have been presented in Fig. 5.

$A(\varphi (\theta ^{\prime} ))$ and $\omega (\varphi (\theta ^{\prime} ),\theta ^{\prime} )$ - amplitude and phase profile functions at each dipole source.

Starting from the inscribed circle in Fig. 5, we obtain the SPP electric field equations for a circular plasmonic lens (CPL), and modify it to an HPL equation by applying geometric constraints elaborated in Fig. 5. Consider the amplitude and phase profiles-

$$A(\varphi (\theta ^{\prime} ))={A}_{o}\,\cos \,\varphi (\theta ^{\prime} );\,\omega (\varphi (\theta ^{\prime} ),\theta ^{\prime} )=\,\pm \,\varphi (\theta ^{\prime} )$$

The positive and negative signs correspond to LCP and RCP incidence, respectively.

In case of circular polarization, the dipole sources are aligned parallel to the radius, hence –

$$\varphi (\theta ^{\prime} )=\theta ^{\prime} ;\,\phi (\theta ^{\prime} )=0$$

In case of RCP illumination

$$\omega (\varphi (\theta ^{\prime} ),\theta ^{\prime} )=-\,\varphi (\theta ^{\prime} )=-\,\theta ^{\prime} $$

$$A(\varphi (\theta ^{\prime} ))={A}_{o}\,\cos \,\phi (\theta ^{\prime} )={A}_{o}$$

The term |ρ − ρ′| represents distance between the source of plasmons and the point of investigation. Therefore, using cosine rule- $|{\rm{\rho }}-{\rm{\rho }}^{\prime} |=\sqrt{{{\rm{\rho }}}^{2}+{r}_{o}^{2}-2{\rm{\rho }}{r}_{o}\,\cos (\theta -\theta ^{\prime} )}$; where r_0_ is the radius of CPL.

Now plugging all the values in $d{E}_{z}(\rho ,\theta ,z)$ and integrating it from 0 to 2π, we get-

1 $${E}_{z}(\rho ,\theta ,z)={A}_{o}{e}^{-{k}_{a}z}{\int }_{0}^{2\pi }{e}^{-j\theta ^{\prime} }{e}^{j{k}_{spp}\sqrt{{{\rm{\rho }}}^{2}+{r}_{o}^{2}-2{\rm{\rho }}{r}_{o}\cos (\theta -\theta ^{\prime} )}}{\rm{d}}\theta ^{\prime} $$

By similar analyses, for linear polarized incident beams we obtain the following equations –

For x-polarized light,

2 $${E}_{z}(\rho ,\theta ,z)\propto {\int }_{0}^{2\pi }\cos \theta ^{\prime} {e}^{j{k}_{spp}\sqrt{{{\rm{\rho }}}^{2}+{r}_{o}^{2}-2{\rm{\rho }}{r}_{o}\cos (\theta -\theta ^{\prime} )}}{\rm{d}}\theta ^{\prime} $$

For y-polarized light,

3 $${E}_{z}(\rho ,\theta ,z)\propto {\int }_{0}^{2\pi }\sin \theta ^{\prime} {e}^{j{k}_{spp}\sqrt{{{\rm{\rho }}}^{2}+{r}_{o}^{2}-2{\rm{\rho }}{r}_{o}\cos (\theta -\theta ^{\prime} )}}{\rm{d}}\theta ^{\prime} $$

Now we’ll modify Eqs (1), (2), (3) for hexagonal plasmonic lens (HPL) by applying the suitable geometric constraints. As presented in Fig. 5, the distance of any point of the hexagon from the centre, for ${\rm{\theta }}^{\prime} \epsilon \,[0,\frac{{\rm{\pi }}}{3}]$, is given by-

$$r={r}_{o}\,\sec (\theta ^{\prime} -\frac{\pi }{6})$$

Since it is a regular hexagon, the same equation will be applicable for the remaining sections i.e. θ′ ∈ [π/3, 2π/3], [2π/3, π], [π, 4π/3], [4π/3, 5π/3], [5π/3, 2π]. Hence, for the HPL, by cosine rule, $|\rho -\rho ^{\prime} |=\sqrt{{{\rm{\rho }}}^{2}+{\{{r}_{o}\sec (\theta ^{\prime} -\frac{\pi }{6})\}}^{2}-2{\rm{\rho }}{r}_{o}\,\sec (\theta ^{\prime} -\frac{\pi }{6})\cos (\theta -\theta ^{\prime} )}\,$. For the section corresponding to ${\rm{\theta }}^{\prime} \epsilon \,[0,\frac{{\rm{\pi }}}{3}]$, *E*_*SPP*_ is expressed as-

$${E}_{z}{(\rho ,\theta ,z)}_{{\rm{\theta }}^{\prime} \epsilon [0,{\rm{\pi }}/3]}={A}_{o}{e}^{-{k}_{a}z}{{\int }_{0}^{\pi /3}{e}^{-j\theta ^{\prime} }e}^{j{k}_{spp}\sqrt{{{\rm{\rho }}}^{2}+{\{{r}_{o}\sec (\theta ^{\prime} -\frac{\pi }{6})\}}^{2}-2{\rm{\rho }}{r}_{o}\sec (\theta ^{\prime} -\frac{\pi }{6})\cos (\theta -\theta ^{\prime} )}}{\rm{d}}\theta ^{\prime} $$

Similarly, we can obtain the corresponding equations for the remaining 5 sections. Using this equation, we have plotted the E-field intensity and phase in MATLAB, as shown in Fig. 3. The scaling behavior is guided by change in *r*_*o*_ and *k*_*SPP*_. As evident from Fig. 3, the analytical plots conform well to the FDTD simulation results.

Similarly, for x-polarized incident beam,

$${E}_{z}{(\rho ,\theta ,z)}_{{\rm{\theta }}^{\prime} \epsilon [0,{\rm{\pi }}/3]}={A}_{o}{e}^{-{k}_{a}z}{{\int }_{0}^{\pi /3}\cos \theta ^{\prime} e}^{j{k}_{spp}\sqrt{{{\rm{\rho }}}^{2}+{\{{r}_{o}\text{sec}(\theta ^{\prime} -\frac{\pi }{6})\}}^{2}-2{\rm{\rho }}{r}_{o}\text{sec}(\theta ^{\prime} -\frac{\pi }{6})\cos (\theta -\theta ^{\prime} )}}{\rm{d}}\theta ^{\prime} $$

For y-polarized incident beam,

$${E}_{z}{(\rho ,\theta ,z)}_{{\rm{\theta }}^{\prime} \epsilon [0,{\rm{\pi }}/3]}={A}_{o}{e}^{-{k}_{a}z}{{\int }_{0}^{\pi /3}\sin \theta ^{\prime} e}^{j{k}_{spp}\sqrt{{{\rm{\rho }}}^{2}+{\{{r}_{o}\sec (\theta ^{\prime} -\frac{\pi }{6})\}}^{2}-2{\rm{\rho }}{r}_{o}\sec (\theta ^{\prime} -\frac{\pi }{6})\cos (\theta -\theta ^{\prime} )}}{\rm{d}}\theta ^{\prime} $$

### Device Scheme

**Figure 6:**
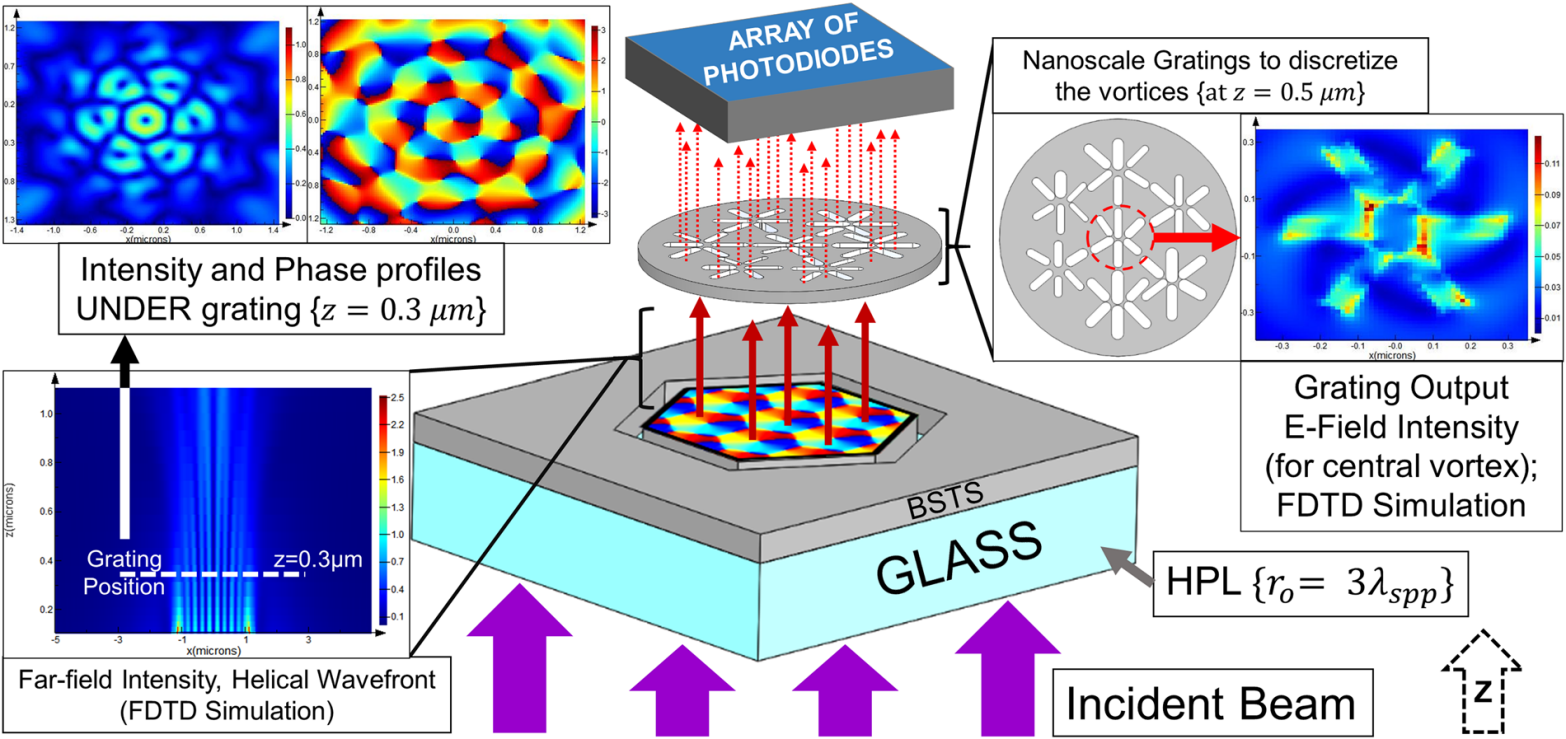
Device scheme based on optical vortices formed by HPL.

Finally, we propose a novel device scheme of information reader and decoder. As stated by Kirilenko *et al*.^17^, theoretically, infinite information can be encoded into optical vortices. Hence, there is a need for sophisticated devices to read and decode this encoded information in such vortices. We propose to use TI like BSTS for decoder device scheme in the present work. Figure 6 presents the proposed scheme to read and decode information transmitted through an electromagnetic wave. A beam with encoded information is incident on the bottom of the device. A scalar vortex pattern, with helical (or equivalent) wave-front is obtained on the top surface of the HPL. Figure 6 presents a lens with ${r}_{o}=3{\lambda }_{spp}$. The emission from the lens acts as an input for a thin metallic film consisting of nano-scale gratings which discretizes the helical wave-front. Here, six radial gratings are used for each optical-vortex, although the number can be increased in accordance with state of the art. The shape of the polarizer gratings can be optimized to control the output polarization of the transmitted optical field, as reported by Li J *et al*.^18^, so as to minimize losses by scattering. Furthermore, the operating distance of this ‘information discretizing film’ will depend on the far-field intensity, though near-field polarizing gratings can also be fabricated. The far-field intensity pattern in z-direction has been presented in the figure. To finally fabricate a feasible device, it is very important to check the far-field intensity of the SPP vortex pattern. The obtained intensity and phase patterns at $z=0.3\,\mu m$ have been shown in Fig. 6. Furthermore, the far-field phase and intensity patterns for E_x_, E_y_ and E_z_ have been presented in Figure S4 in supplementary information. We can see that the vortex pattern and intensity distribution are maintained up to this height, which provides a comfortable operating distance for the patterned discretizing film. Further, we calculated (FDTD) the output intensity from set of 6 radial gratings placed corresponding to each scalar vortex formed in HPL, as presented in Fig. 6.

The equation of z-direction electric field of the optical vortex element can be obtained by considering superposition of optical elements of various angular phases (φ). Assuming the optical vortex to be a resultant of n-optical elements, the following equation is obtained by superposition, as presented by Kirilenko *et al*.^17^.

4 $${E}_{z}(r,\phi )={\sum }_{k=1}^{n}{A}_{k}{e}^{ik\phi }$$

E_z_ = electric field as a function of radius (r) from singular point of vortex, and phase of superposing optical element (φ) forming the vortex; A_k_ = Normalized Amplitude of the optical element

The scheme presented in Fig. 6 contains six gratings above the vortex to discretize information. Which means that n = 6 optical elements of variable intensity can be obtained above the gratings. For this case, the phase of optical elements, φ, can be taken as a product of π/3. Hence corresponding to the 6 gratings, the respective phases of the optical elements transmitted are φ = 0, π/3, 2π/3, π, 4π/3, 5π/3, corresponding to k = 1, 2, 3, 4, 5, 6 in Eq. 4.

**Figure 7:**
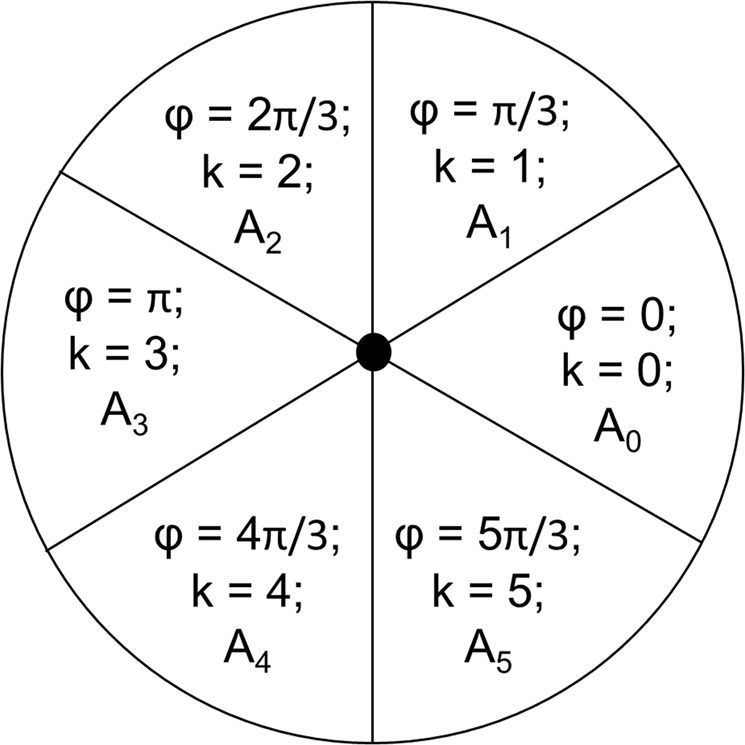
Phase and amplitude of vortex elements transmitted through the 6 gratings above the vortex (particular case with n = 6).

Finally, the discretized packets of waves transmitted through the polarizing gratings will be detected by photodiodes whose position will correspond to the gratings in the discretizing film^19,20^. The photodiodes will provide the amplitude of transmission from the gratings as the electric signal obtained will be proportional to beam intensity. This will allow us to store the amplitude information corresponding to each φ, as presented in Fig. 7. Based on these variable amplitudes (A_1_, A_2_…) at the different phases (φ_1_, φ_2_…) information can be decoded from the vortex patterns. Furthermore, the number of gratings, and hence, n, can be increased to add more terms to the superposition in Eq. 4. The decoding process will work as a reverse of the encoding method of singular vortex reported earlier^17^.

In the Fig. 6, we have presented a hexagonal lens of 3λ_spp_ radius. An optical lattice of 7 vortices is formed on the lens. The current scheme utilizes 6 polarizer gratings per vortex, which correspond to 6 discretized elements. Hence, from the 7 vortices, we get 42 packets of information, which can be combined to decode the overall pattern. Similarly, owing to the parabolic ‘scaling behavior’ validated in the preceding sections, for 4λ_spp_ radius, there will be 19 vortices, corresponding to 114 discretized optical elements. For 5λ_spp_ radius, there will be 37 vortices, resulting 222 discretized optical elements. The size of the $3{{\rm{\lambda }}}_{{\rm{spp}}},4{{\rm{\lambda }}}_{{\rm{spp}}},5{{\rm{\lambda }}}_{{\rm{spp}}}$ radii lenses are 2.4 µm, 3.2 µm and 4 µm. The number of optical elements considered in this example is n = 6, which can be further increased in accordance with the state of the art.

## Conclusion

In conclusion, we have presented a new device scheme for optical memories based on TI. These hexagonal plasmonic lens (HPL) encrypted TI based optical memories have shown 2-D optical lattices of scalar vortices. Furthermore, we have shown the scaling path of these optical devices by numerical simulations (FDTD), validated by theoretical calculations. Advantage of scaling in optical devices without any additional processing shows the promise of this technology for future devices.

## Supplementary information

Supplementary Information
