# Supplementary material for: Design of Scalable Optical Decoder based on Hexagonal Plasmonic Modes induced on Topological Insulator Surface States
Source: Sci Rep. 2019 Jun 24;9:9190. doi: 10.1038/s41598-019-45607-z (PMC6591422; doi:10.1038/s41598-019-45607-z)
Supplement: Supplementary file 1 — Supplementary Information [file 41598_2019_45607_MOESM1_ESM.docx]

**Design of Scalable Optical Decoder based on Hexagonal Plasmonic Modes induced on Topological Insulator Surface States**

Siddharth Srivastava, Priyanshu Jain and Tanmoy Maiti*

Plasmonics and Perovskites Laboratory, Dept. of Materials Science and Engineering,

IIT Kanpur, U.P. 208016, India.

SUPPLEMENTARY INFORMATION


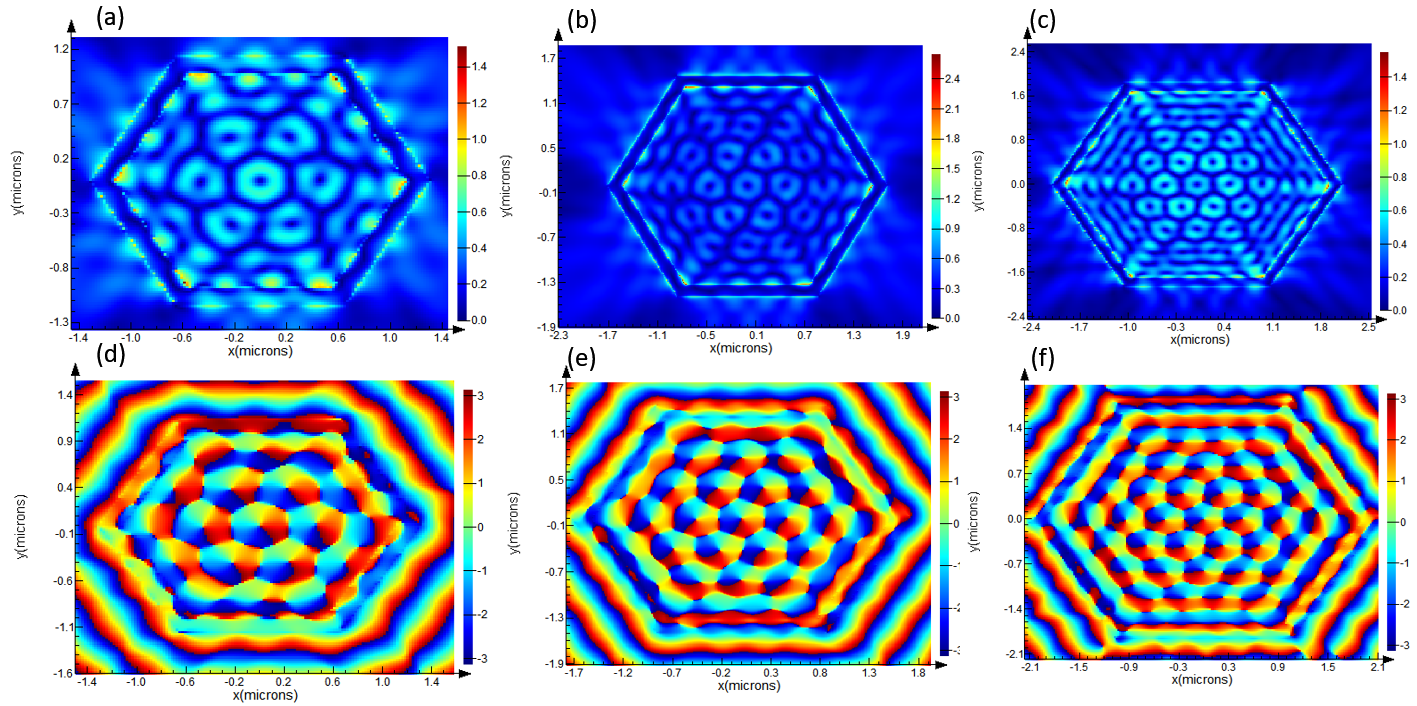


***Figure S1*** *FDTD simulation of Electric field (E_z_) distribution (a,b,c) and phase distribution (d,e,f) for Hexagonal Plasmonic Lens under LCP illumination with varying radii,* $r_{o}= {3\lambda}_{spp},{4\lambda}_{spp},{5\lambda}_{spp}$*, for BSTS/GLASS*

*
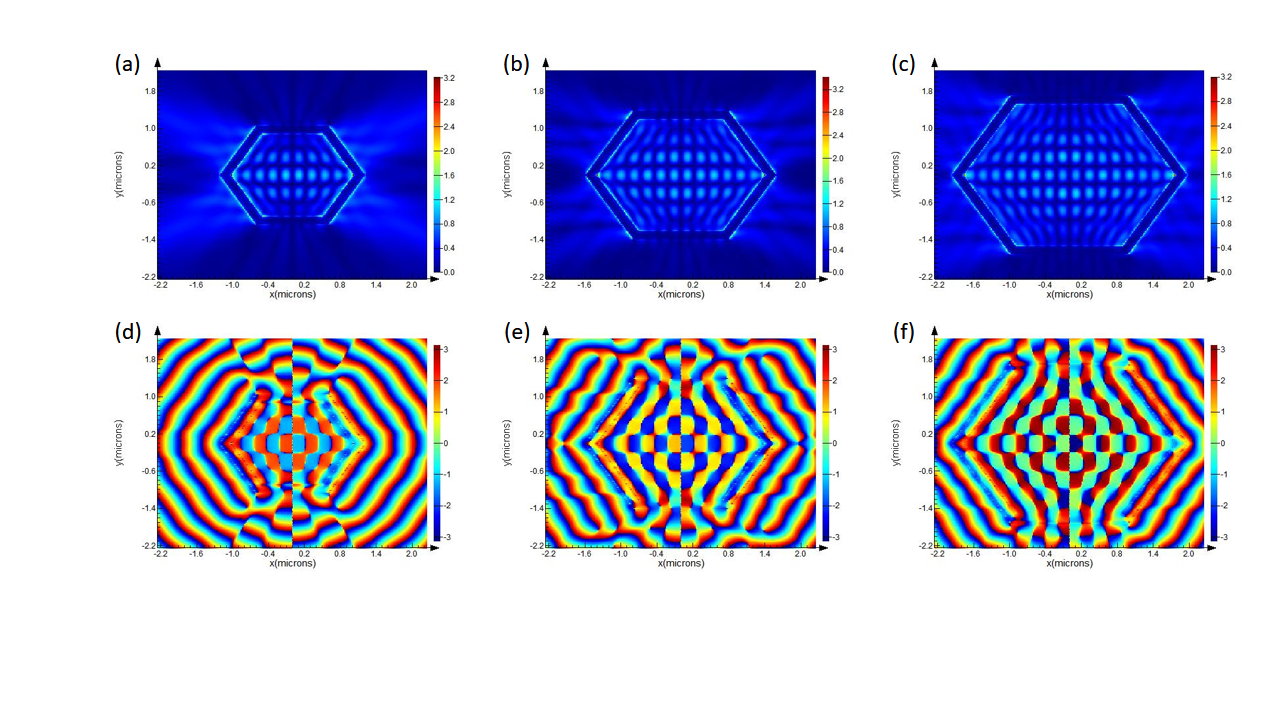
*

***Figure S2*** *FDTD simulation of Electric field (E_z_) distribution (a,b,c) and phase distribution (d,e,f) for Hexagonal Plasmonic Lens under x-polarized illumination with varying radii,* $r_{o}= {3\lambda}_{spp},{4\lambda}_{spp},{5\lambda}_{spp}$*, for BSTS/GLASS*

*
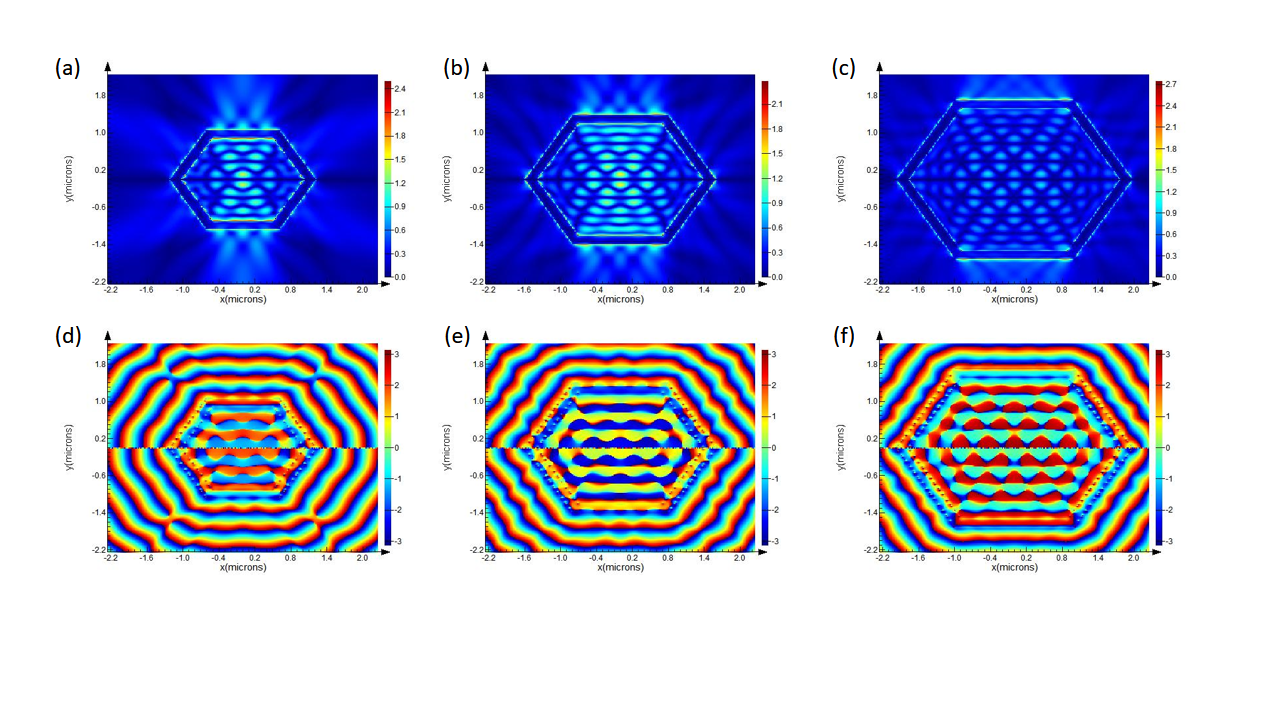
*

***Figure S3*** *FDTD simulation of Electric field (E_z_) distribution (a,b,c) and phase distribution (d,e,f) for Hexagonal Plasmonic Lens under y-polarized illumination with varying radii,* $r_{o}= {3\lambda}_{spp},{4\lambda}_{spp},{5\lambda}_{spp}$*, for BSTS/GLASS*


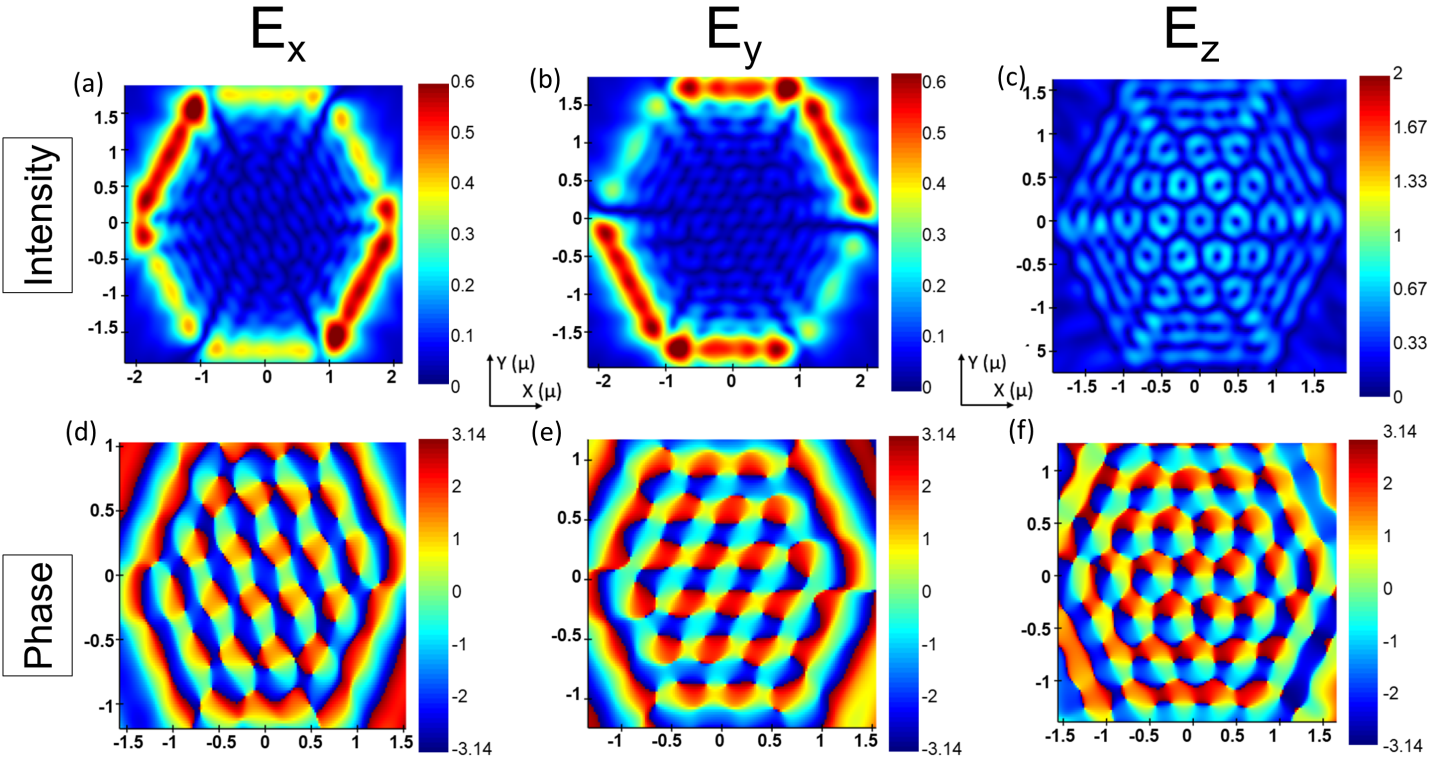


**Figure S4.** FDTD simulation of Far-Field Electric field and phase components for BSTS/Glass Hexagonal Plasmonic Lens under RCP illumination with radius, $r_{o}={5\lambda}_{\mathrm{spp}}$.
